# Identification of Predictive Early Biomarkers for Sterile-SIRS after Cardiovascular Surgery

**DOI:** 10.1371/journal.pone.0135527

**Published:** 2015-08-11

**Authors:** Sandra Stoppelkamp, Kujtim Veseli, Katharina Stang, Christian Schlensak, Hans Peter Wendel, Tobias Walker

**Affiliations:** Department of Thoracic, Cardiac and Vascular Surgery, University Hospital Tuebingen, Tuebingen, Germany; Yale University, UNITED STATES

## Abstract

Systemic inflammatory response syndrome (SIRS) is a common complication after cardiovascular surgery that in severe cases can lead to multiple organ dysfunction syndrome and even death. We therefore set out to identify reliable early biomarkers for SIRS in a prospective small patient study for timely intervention. 21 Patients scheduled for planned cardiovascular surgery were recruited in the study, monitored for signs of SIRS and blood samples were taken to investigate biomarkers at pre-assigned time points: day of admission, start of surgery, end of surgery, days 1, 2, 3, 5 and 8 post surgery. Stored plasma and cryopreserved blood samples were analyzed for cytokine expression (IL1β, IL2, IL6, IL8, IL10, TNFα, IFNγ), other pro-inflammatory markers (sCD163, sTREM-1, ESM-1) and response to endotoxin. Acute phase proteins CRP, PCT and pro-inflammatory cytokines IL6 and IL8 were significantly increased (p<0.001) at the end of surgery in all patients but could not distinguish between groups. Normalization of samples revealed significant increases in IL1β changes (p<0.05) and decreased responses to endotoxin (p<0.01) in the SIRS group at the end of surgery. Soluble TREM-1 plasma concentrations were significantly increased in patients with SIRS (p<0.01). This small scale patient study could show that common sepsis markers PCT, CRP, IL6 and TNFα had low predictive value for early diagnosis of SIRS after cardiovascular surgery. A combination of normalized IL1β plasma levels, responses to endotoxin and soluble TREM-1 plasma concentrations at the end of surgery are predictive markers of SIRS development in this small scale study and could act as an indicator for starting early therapeutic interventions.

## Introduction

Systemic inflammation is a common response in critically ill patients and a known side-effect after surgery [1,2]. Initially, inflammatory reactions have not been characterized consistently, but with the definitions of the American College of Chest Physicians/Society of Critical Care Medicine (ACCP/SCCM) consensus conference [3] a better classification of the various stages of infectious and non-infectious inflammation and their severity became available and widely accepted. The nature and causes of those inflammatory responses have been identified more clearly in the last decades, especially with advances in immunology particularly, the discovery of cytokine mediators. Although the underlying causes are manifold, ranging from invasion of different pathogens over hypoxia to cell injury, the host response can be attributed to the overwhelming uncontrolled activation of the innate and adaptive immune system [1,4]. The severity and progress of the disease thus not only depends on the strength of the insult but is also a reflection of the patient’s individual immune response to danger signals. In contrast to other elective surgeries, patients undergoing cardiovascular surgery often are elderly and represent with comorbidities and a weakened general condition. Those patients are therefore especially at risk of complications such as systemic inflammatory response syndrome (SIRS). This generic term encompasses sterile inflammation as well as sepsis (that is SIRS with confirmed bacteremia) and is defined by meeting two or more of the following criteria: 1) a temperature of above 38°C or below 36°C, 2) a heart rate over 90 beats/min, 3) a respiratory rate above 20 breaths/min or decreased paCO_2_ below 32 mmHg, 4) white blood cell count of over 12 000 cells/mm^3^ or under 4000 cells/mm^3^ or more than 10% immature neutrophils [3]. In this current paper SIRS is used to describe the state of sterile inflammation without positive blood culture.

Especially in cardiac surgery with cardiopulmonary bypass (CPB) systemic inflammation is a well described phenomenon (reviewed in [5]), attributed in part to predisposing factors of the patient and the surgery insult [6]. During surgery, the complex regulation of hemostatic parameters (coagulation, fibrinolytic and complement system, leukocytes, endothelial cells and platelets) is disturbed [7] calling for counter regulatory measures. Particularly in CPB surgery, it is of utmost importance to ensure hemocompatibility, but also test for contaminations that may induce similar hemostatic disturbances as incompatible materials [8]. While most of the patients showing signs of inflammation after surgery due to trauma and other stressors such as ischemia reperfusion injury, mechanical shear stress, hemodilution, hypothermia, cellular activation upon contact with extracorporeal circulation (ECC), only a minority of patients develop SIRS or severe complications [9]. The severity of these complications, however, is associated with deteriorating outcome: mortality rates increase from SIRS (10%) over sepsis (20%) to severe sepsis (40%) and septic shock (80%) [10].

Data on SIRS epidemiology, risk factors and outcome are not as abundant as for sepsis and even those are highly variable depending on the data collection (study design, hospital ward, subjects, medical condition etc.). In Germany, the overall proportion of severe sepsis and septic shock in intensive care units (ICU) is currently stated as 11% with a 90-day-death rate of 54% [11,12]. There was however no differentiation between sepsis and SIRS in these studies. Brun-Buisson describes the incidence of SIRS in ICU patients in Europe as over 80% [13]. These contrasting numbers might on the one hand arise from differences between countries but more likely are due to different comorbidities and general condition of the patients as well as the type of ICU and medical field. This is illustrated by comparative data published for sepsis prevalence in Europe with 37.4% of sepsis patients in ICU but only 18% after elective surgery [14]. Numbers for cardiovascular elective surgery were not specifically mentioned but it is well known that CPB surgery and in particular contact to foreign surfaces in ECC induces inflammatory reactions that can culminate in coagulopathies (disseminated intravascular coagulation–DIC) and organ dysfunctions [15]. Initially, evolving multiple organ failure and death out of SIRS was disputed, but with better understanding of molecular mechanisms of the innate immune system, it is recognized that a strong host response to an insult can manifest in patients as SIRS or even shock, multiple organ dysfunction syndrome (MODS) or multiple organ failure (MOF) [16]. After or alongside the pro-inflammatory state of SIRS, the host immune system often counter regulates with anti-inflammatory mechanisms. If not balanced, those in turn can result in the compensatory anti-inflammatory response syndrome (CARS) or measurable immune paralysis [17] accounting for the development of opportunistic infections [18,19]. The earlier a potential SIRS/sepsis is diagnosed and treatment initiated, the better the outcome, especially for sepsis and septic shock [20]. Therefore, early and precise diagnosis is crucial for successful treatment. The majority of studies investigated the incidence, diagnosis and treatment of sepsis but not as many data exist for SIRS events after cardiovascular surgery.

Here, we set out to identify early biomarkers for SIRS especially after cardiovascular surgery and involving ECC. With the help of eight consecutive time points before and after surgery, we sought to establish a combination of biomarkers predicting the development of SIRS available for clinical decision in the early post-operative period or at early hours in the ICU in order to timely counteract the dysregulation of the immune system.

## Materials and Methods

### Patients

The study was approved by the local institutional review board (University Hospital Tuebingen Ethics committee; project approval number: 231/2013BO2) and conducted in accordance with the declaration of Helsinki. 21 patients scheduled for elective cardiovascular surgery were enrolled and gave their written informed consent. Table 1 shows the patient characteristics. All patients received the same anesthetics (Midazolamhydrochlorid, Sufentanil, Sevofluran) and perioperative antibiotic treatment (Cefazolin 2g bolus i.v.). As can be seen in Table 2, patients presented with comorbidities common to most cardiac patients such as hypertension, hyperlipidaemia and diabetes. Those patients received standard medication for their conditions: angiotensin-converting-enzyme inhibitors for arterial hypertension, statins for hyperlipidaemia. Besides the continuous monitoring of the clinical parameters (temperature, heart and respiratory rates, blood pressure, blood count, C-reactive protein (CRP) and procalcitonin (PCT) values blood samples were regularly taken for later analyses. Upon surgery, patients were observed for signs of systemic inflammatory response syndrome (SIRS) and divided into two groups: aseptic controls and SIRS. Inclusion criteria for SIRS were two or more of the following: temperature >38°C or <36°C, heart rate >90 beats/min, respiratory rate >20 breaths/min or p_a_CO_2_ <32 mmHg, white blood cell count of >12.000 cells/mm^3^ or <4000 cells/mm^3^. At completion of treatment, all collected patient data were analyzed to determine SIRS and aseptic control groups. Since only five patients showed signs of SIRS, five aseptic patients out of 16 were chosen for comparison to equalize group sizes; patient characteristics of these two groups are shown in Table 2. Stored blood and plasma samples of those 10 patients were then investigated for cytokine levels, inflammatory / sepsis biomarkers and general stimulation ability at eight different time points during hospitalization.

**Table 1:** Characteristics of all recruited patients. Shown are means of the criteria ± SD.

Criterion	Patient numbers
n =	21
Age (years)	69.8 ± 10.6
Gender (male / female)	19 / 2
Height (cm)	172.2 ± 5.6
Weight (kg)	81.8 ± 14.3
Hospitalization (days)	15.4 ± 10.7
OP duration (min)	251.5 ± 55.3

**Table 2:** Data of selected study subjects (5 per group aseptic (NS) and SIRS).

Patients	NS	SIRS
n =	5	5 (4; 1death day 2)
Age (years)	69.8 ± 10.6	61.4 ± 15.9
Gender (male/female)	3 / 2	5 / 0
Height (cm)	172.2 ± 5.6	172.2 ± 5.4
Weight (kg)	81.8 ± 14.3	96.0 ± 19.9
Hospitalization (days)	10.0 ± 2.9	29.5 ± 7.8 *
Duration of surgery (min)	249.0 ± 75.1	284.6 ± 56.1
**Comorbidities**		
Adipositas BMI ≥25–<30	20%	60%
BMI ≥30–<35	20%	20%
BMI ≥35–<40	20%	
BMI ≥40		20%
Diatetes mellitus		20%
Diabetes type 2	20%	
(Arterial) hypertension	40%	60%
Hyperlipidaemia	20%	60%
Peripheral arterial occlusive disease	20%	
COPD		20%
Steatosis hepatis		20%
**Therapeutic regimens**		
elective coronary bypass surgery/revascularization	40%	100%
aortic valve replacement	20%	
mitral valve surgery	40%	

Data are divided into the two groups, aseptic and SIRS and presented as mean ± SD

or percentage of patients per group, respectively.

* p <0.05; Mann Whitney U-test.

COPD = chronic obstructive pulmonary disease; day 2 = two days post surgery.

### Plasma and Blood Sampling

Blood was collected from all patients on the day of admission to the hospital, at the beginning and end of the surgery and on days 1, 2, 3, 5 and 8 post surgery either by venipuncture or from a central venous catheter in heparinized monovettes (19 I.U./ml, Sarstedt, Germany) and EDTA monovettes (EDTA-K 1.6 mg/ml, Sarstedt, Germany). One part of the blood samples was sent to the central laboratory facility (University Hospital Tuebingen) for analysis of leukocyte counts, C-reactive protein (CRP) and procalcitonin (PCT) levels. Another set of monovettes was used within four hours of collection to prepare cryopreserved blood and plasma for later analyses of cytokine levels and other biomarkers. Plasma was obtained from EDTA-K monovettes by centrifugation at 2500 g for 20 min at 4°C, shock-frozen in 200 μl aliquot sizes in liquid nitrogen and stored at -80°C. Cryopreserved blood was generated by acclimatizing the heparinized blood at 4°C and subsequent slow addition of 9.1% DMSO. The blood/DMSO mixture was slowly frozen down to -80°C at a cooling rate of approximately 1°C/min (Nalgene Mr. Frosty Isopropanol container; Thermo Scientific, Germany) and stored in liquid nitrogen until use.

### Cytokine assessment

Cytokine levels were determined with a multiplex immunoassay (Human High Sensitivity Cytokine Luminex Performance Assay; R&D Systems, Germany) according to manufacturer’s instructions. Briefly, frozen plasma samples were diluted 1:2 with Calibrator Diluent RD6-40 and run with polystyrene microparticle beads in a Bioplex 200 (BioRad, Germany). Seven analytes were assayed simultaneously with the following limits of quantification (LLOQ): IL1β: 0.34 pg/ml; IL-2: 0.58 pg/ml; IL-6: 0.92 pg/ml; IL-8: 0.76 pg/ml; IL-10: 0.47 pg/ml; IFNγ: 0.30 pg/ml; TNFα: 0.78 pg/ml.

### ELISA of inflammatory biomarkers and cell-free DNA quantification

Plasma levels of three soluble inflammatory markers were determined via ELISA (Trillium Diagnostics, Maine, USA, distributed by IQ Products, Groningen, Netherlands) based on a chromogenic substrate (TMB). All assays were performed according to manufacturer’s instructions with the following dilutions of freshly thawed plasma samples: human endothelial cell-specific molecule (ESM-1, endocan) 1:2 aseptic samples, 1:6 SIRS samples; soluble CD163 (MACRO163) 1:500 and human Triggering Receptor Expressed on Myeloid Cells 1 (sTREM-1) 1:5. Substrate intensities were quantified by Eon Reader with Gen5 Software (Biotek, Bad Friedrichshall, Germany). The software determined the plasma concentrations with the help of protein standard dilutions comprised in the kits. Patient plasma levels of cell-free DNA were quantified with the cfDNA-Quant kit (Trillium Diagnostics, Maine, USA) according to manufacturer’s instructions with a dilution of 1:5. Relative fluorescent intensities were determined with a fluorescent plate reader (Mithras LB940, Bad Wildbad, Germany) and gained intensities were transformed into plasma concentrations with the help of an analysis template provided by the manufacturer.

### Monocyte Activation Test (MAT)

Cryopreserved whole blood of each patient sample was thawed for 90 sec in a 37°C water bath and directly diluted 1:5 with medium (RPMI 1640, Biochrom AG, Germany) without further supplements. A 2-fold series dilution of Endotoxin (WHO 3^rd^ international standard endotoxin; NIBSC, UK) in medium was prepared at concentrations of 0.125 to 2 EU/ml. A mixture of equal volumes medium and endotoxin dilution / blank (together 40 μl) were combined with 200 μl of the diluted blood in the Cellstar flat bottom pyrogen-free 96-well-plates (Greiner Bio-One GmbH, Frickenhausen, Germany). As assay control, PyroDetect Cryoblood (Merck Millipore, Germany) was used on every 96-well-plate to guarantee valid and consistent performance. The endotoxin-stimulated cryoblood was incubated at 37°C 5% CO_2_ for 8 h. After the incubation, plates were sealed with adhesive foil and frozen at -20°C. The ability of patient blood samples to be stimulated by endotoxin was observed by analysis of pro-inflammatory IL-1β levels via ELISA with matched antibody pairs (R&D Systems, Germany). Briefly, 100 μl of the endotoxin-stimulated cryoblood samples was transferred to coated (50 μl anti-human monoclonal IL-1β MAB601; 4 μg/ml) ELISA plates (flat-bottom 96-well Nunc MaxiSorp plates). After a 2-h-incubation step at room temperature, the plates were washed 3x with 250 μl/well PBS (Biochrom AG, Germany) 0.05% Tween20 (Merck Millipore, Germany) and 50 μl biotinylated detection antibody (200 ng/ml anti-human IL-1β-biotin BAF201) was added for further 2 h at room temperature. Unbound antibody was removed by washing (3x) and 100 μl/well streptavidin-peroxidase solution (R&D Systems) was added and incubated for 30 min at room temperature. Bound IL-1β cytokine was visualized by addition of substrate (3,3´,5,5´tetramethylbenzidine/ H_2_O_2_; R&D Systems). After approximately 11 min the reaction was stopped by adding 50 μl/well 1 M H_2_SO_4_ and absorbance read at 450 nm with a reference wavelength of 630 nm (Eon Microplate Reader with Gen5 Software, BioTek, Germany). A 2-fold dilution series of human recombinant IL-1β protein was run on each ELISA plate to calculate IL-1β protein concentrations from the obtained absorbance values. Calculations (blank subtraction and protein concentrations) were performed by the Gen5 Software (BioTek, Germany).

### Statistical analyses and calculations

Obtained plasma concentrations of all assays were also used to calculate the fold change over time of hospital stay. For this calculation, each sampling time point was normalized to the corresponding value of the admission day (time point x/admission day).

Statistical analyses were performed with the GraphPad PRISM Software Version 5.01 (GraphPad Software, Inc., USA). Since a small sample size of 5 patients per group was used, nonparametric Kruskal-Wallis test with Dunn’s multiple parameter posttest for selected data were chosen. Values of p ≤ 0.05 were considered as being significant, values of p > 0.05 were considered as being not significant (n.s.).

## Results

### Selection of patient groups

**Fig 1:**
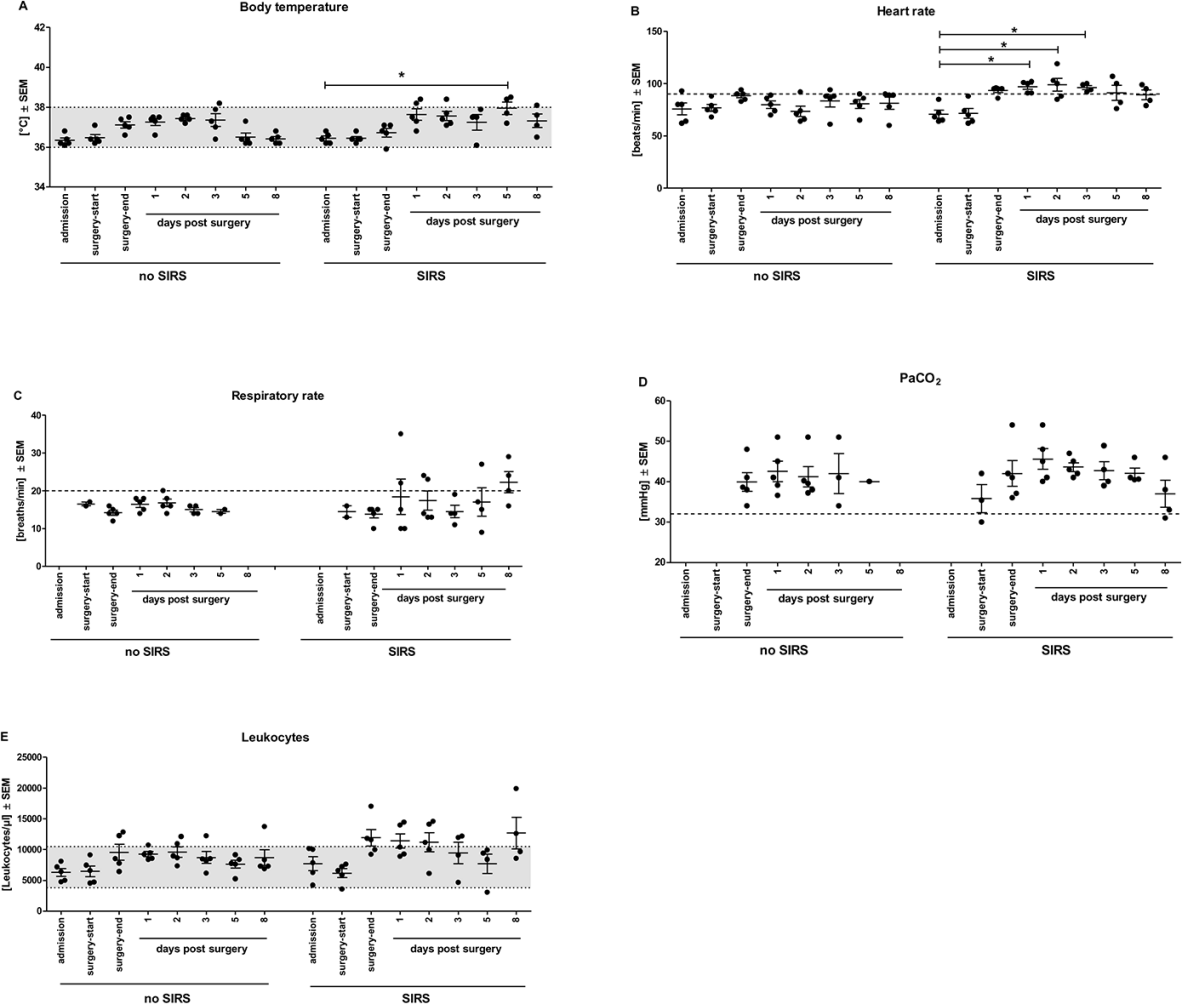
SIRS parameters of selected study patients. (A) Body temperature, (B) heart rate, (C) breathing rate, (D) PaCO_2_ and (E) leukocyte counts are presented as means ± SEM per study group. Dotted lines indicate threshold values to pathological levels, shaded areas between dotted lines indicate the physiological ranges. * p < 0.05 Kruskal-Wallis with Dunn’s multiple comparison post-hoc test for selected data pairs.

The study was designed to monitor patients over the course of eight days pre- and post-surgery in order to find early biomarkers for SIRS. The pre-assigned time points were: 1) day of admission, 2) start of surgery, 3) end of surgery, 4–8) days 1, 2, 3, 5 and 8 post surgery. Patients undergoing elective cardiovascular surgery were recruited after ethical approval and monitored in addition to the usual medical parameters for signs of SIRS (temperature, leukocyte counts, respiratory and heart rates) and early response proteins C-reactive protein (CRP) and procalcitonin (PCT) (see Fig 1 and S1 Fig, respectively). Over eight time points, additional blood samples were withdrawn to store plasma and cryopreserved blood. Special attention was paid to uniform sampling and storage by using one kind of blood collection tubes throughout and previously well trained personnel for blood sampling and processing. Snap freezing of plasma samples in small aliquots ensured usage of pristine patient material for each test. In addition, assays to determine plasma proteins were from the same batch. This was undertaken to prevent possible variations in samples unrelated to the study subject but induced by handling [21]. At completion of recruitment and sampling, patient data were analyzed for signs of SIRS according to the official guidelines of the ACCP/SCCM Consensus Conference [3]. Five patients of the cardiac surgery group were identified to match those criteria; five other patients from the same cardiac surgery group without signs of SIRS were chosen as control group to match group sizes. The control group comprised of patients with similar age, weight, BMI, comorbidities and surgery with ECC but overall lower hospitalization times (see Table 2).

### Plasma levels of acute phase proteins alone have low predictive value for SIRS

As an initial approach to identify early biomarkers for SIRS after cardiovascular surgery, commonly used pro-inflammatory cytokines and biomarkers for sepsis were identified in the two patient groups. Although all sampled time points were investigated, a special attention was given to the first time points (end of surgery and day 1 post surgery) for early intervention purposes. CRP increased readily at days 1 to 3 post surgery, but was similar for both groups (see S1A Fig). Similarly, PCT was elevated, especially at day 1 post surgery, where the increase was significant compared to values at admission (S1B Fig). This increase however, was not different between the two groups and therefore neither CRP nor PCT can indicate early SIRS after cardiovascular surgery. Group means and standard deviations are summarized in Table 2. In addition to those two very common protein markers, pro-inflammatory cytokine plasma concentrations were determined with a multiplex cytokine assay (see S2 Fig). Seven cytokines were quantified, but plasma levels of IL2 and IFNγ were mostly below the quantification limit and are not shown here. The other measured cytokines (IL1β, IL6, IL8, IL10, TNFα) showed significant elevations for IL6 (S2B Fig) and IL8 (S2C Fig) at the end of the surgery, but again those increases were seen for both SIRS and control group. The other measured cytokines did not reveal any characteristic pattern to distinguish early signs of SIRS.

### Normalizing cytokine levels to individual’s basal values can indicate early phases of SIRS

**Fig 2:**
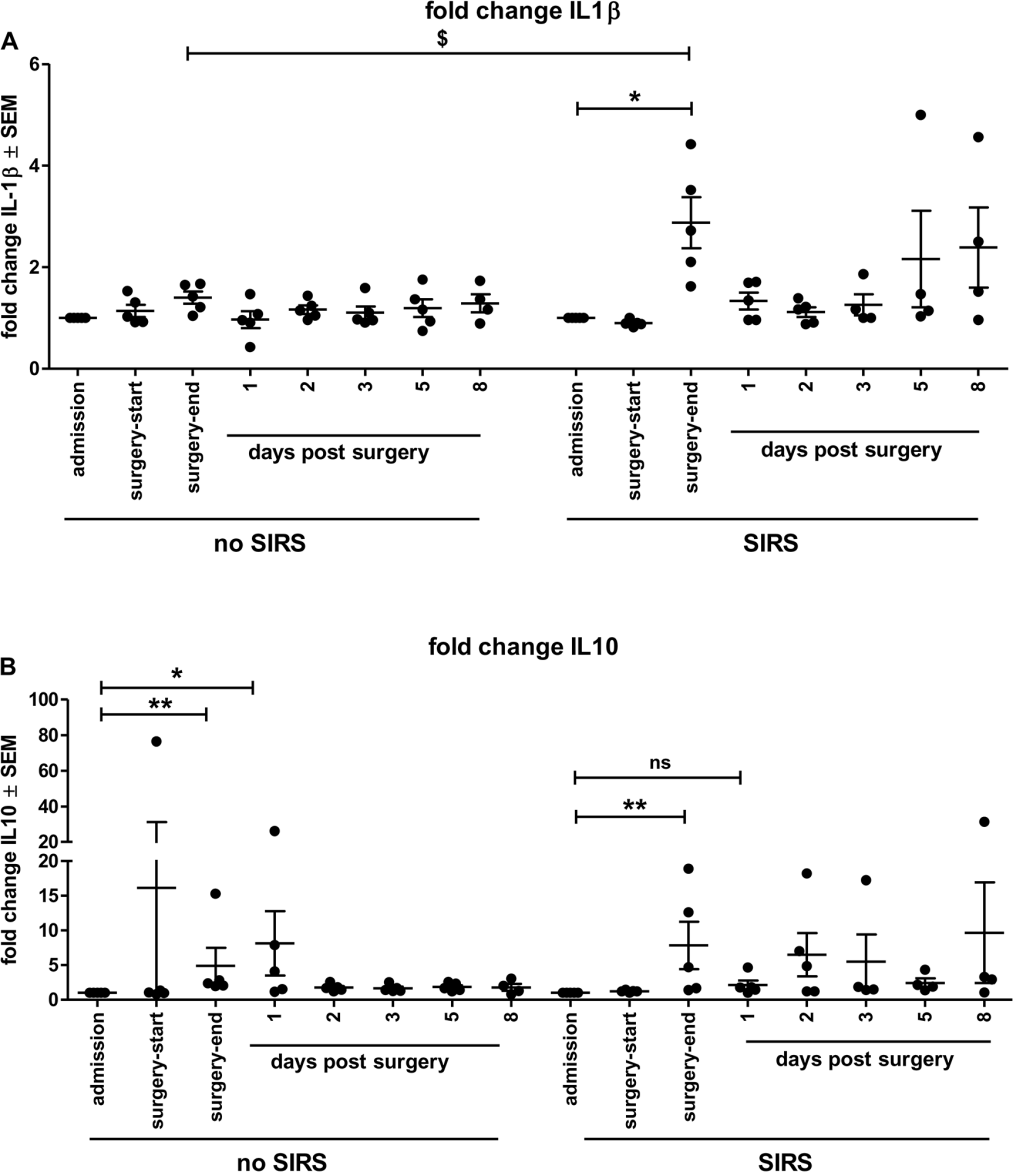
Normalized changes in IL1β and IL10 plasma cytokine levels have potential predictive value for early recognition of SIRS. Shown are fold changes ± SEM in plasma concentrations of (A) IL1β and (B) IL10 per patient, normalized to the day of admission, over the collected time points. * p < 0.05; ** p < 0.01; *** p < 0.001 Kruskal-Wallis with Dunn’s multiple comparison post-hoc test for selected data pairs. $ p < 0.05 Mann Whitney U-test.

Looking more closely at the individual cytokine response pattern rather than only group means revealed a distinct increase at the end of surgery in the SIRS group for IL1β that was not present in the control group. Therefore, all cytokine responses were further normalized to the individual values at admission to the hospital. This calculation to indicate fold changes in cytokine secretions supposedly eliminates individual variances and more clearly shows common response trends. Such individual variances are larger in IL1β responses than in IL6 responses after endotoxin stimulation in healthy subjects [22]. As can be seen in Fig 2A, the SIRS group showed a significant fold increase (p < 0.05) in IL1β expression that was not obvious in the control group. Moreover, IL10 expression changes were also significantly elevated at the end of surgery and one day post-surgery in the control group (Fig 2B) but in the SIRS patients, this difference was only seen at the end of surgery. Therefore, normalized expression changes of IL1β at the end of surgery in combination with those of IL10 at the end of surgery and one day after surgery potentially distinguish between patients in risk of developing SIRS and those that recover normally from surgery.

### Endocan, cfDNA and sTREM-1 are probable additional early biomarkers for SIRS

**Fig 3:**
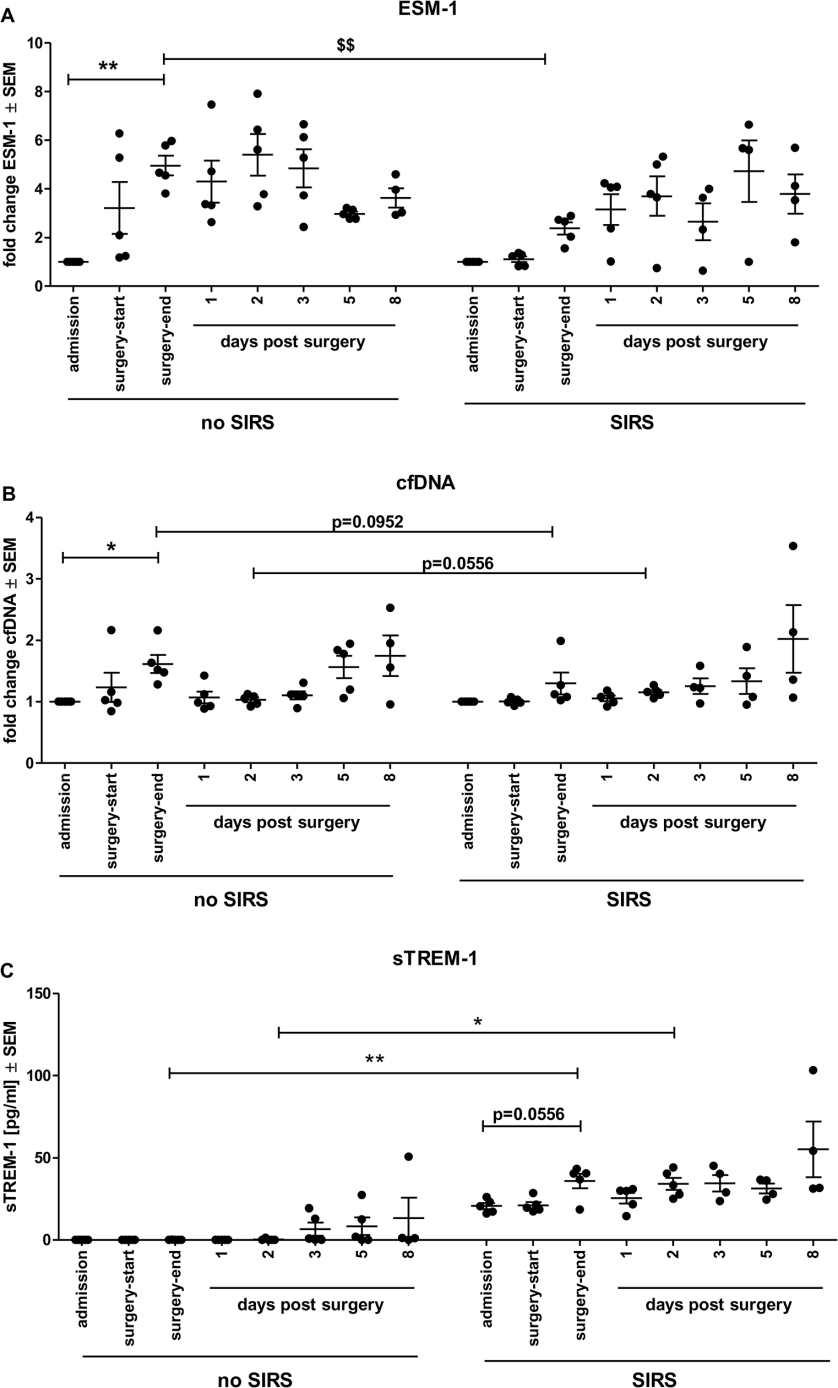
Normalized plasma levels of endocan and cfDNA rise significantly slower whereas soluble TREM-1 plasma levels are upregulated at early time points in SIRS patients. (A) Fold changes in endocan (ESM-1) ± SEM plasma levels of individual patients normalized to the corresponding plasma levels at admission. The fold increase is significantly lower in SIRS group than in control group ** p < 0.01 Kruskal-Wallis with Dunn’s multiple comparison post-hoc test for selected data pairs; $$ p < 0.01 Mann Whitney U-test. (B) Individual plasma levels of cfDNA ± SEM (expressed as fold changes over admission value) tend to increase during surgery but the rise is lower in SIRS than in the control group. * p < 0.05 Kruskal Wallis with Dunn’s multiple comparison post hoc test for selected pairs. (C) Soluble human triggering receptor expressed on myeloid cells-1 (sTREM-1) ± SEM plasma levels are significantly increased in SIRS patients at the end of surgery and day 2 after surgery compared to the control group. * p < 0.05; ** p < 0.01 Kruskal-Wallis with Dunn’s multiple comparison post hoc test for selected data pairs. A trend to an increased expression at the end of surgery within the SIRS group is also seen. p = 0.0556 Mann Whitney U-test.

Besides the mentioned acute phase proteins (cytokines, CRP, PCT) other plasma proteins are currently under investigation for their potential to indicate sepsis or inflammatory states. Here, we focused on soluble plasma proteins that have been described to increase during the course of sepsis (sCD163 and sTREM-1) and as result of inflammatory conditions (endocan/ESM-1). In addition to those soluble proteins, the amount of circulating cell-free DNA (cfDNA) was analyzed as marker for neutrophil activation. CD163, a surface molecule involved in regulation of inflammation, can be shed and is detectable in plasma and other body fluid compartments [23,24]. The expression of soluble CD163 was only slightly elevated in SIRS patients compared to control group (S3B Fig) and also normalization to the admission day did not yield significant differences but an increasing trend is visible (S3D Fig). Endocan, cfDNA and sTREM-1 on the other hand were all differentially regulated in SIRS and control group (Fig 3A–3C). While endocan was elevated after surgery in the control group this trend was not observed in SIRS group (S3A Fig). Moreover, when normalizing the expression to the admission day, the increase was significantly lower in SIRS than in the control group (Fig 2A). The same trend for elevated levels between the groups was observed for cfDNA. At the end of surgery cfDNA levels were significantly elevated in the control group but not in SIRS group (S3C Fig). This difference became slightly more obvious by normalizing the data to the admission day (Fig 3B). Because association of early SIRS with endocan and cfDNA are based mainly on the lack of elevation at the end of surgery, the predictive values of these two markers for sole detection of early SIRS are therefore limited, but these markers can nevertheless serve as additional guidance towards the development of SIRS after cardiovascular surgery. sTREM-1 plasma levels were generally higher in SIRS than in the control group (Fig 3C). This increased expression was significant at the end of surgery (p < 0.01) and on day 2 post surgery (p < 0.05). Within the SIRS group, the sTREM-1 concentrations also showed a trend towards higher expression at the end of surgery compared to the admission day (p = 0.0556). Hence, elevated plasma concentrations of sTREM-1 are a suitable marker to predict developing SIRS conditions after cardiovascular surgery. With the relatively small group sizes a definite cut-off value for sTREM-1 levels to predict SIRS cannot be stated, but would need verification in a larger cohort. A significantly increased value in this study was detected at a mean sTREM-1 level of 35.0 pg/ml and 33.8 pg/ml (p < 0.01 and p < 0.05, respectively).

### Decreasing responses to an LPS challenge in the MAT indicates vulnerability to develop SIRS

**Fig 4:**
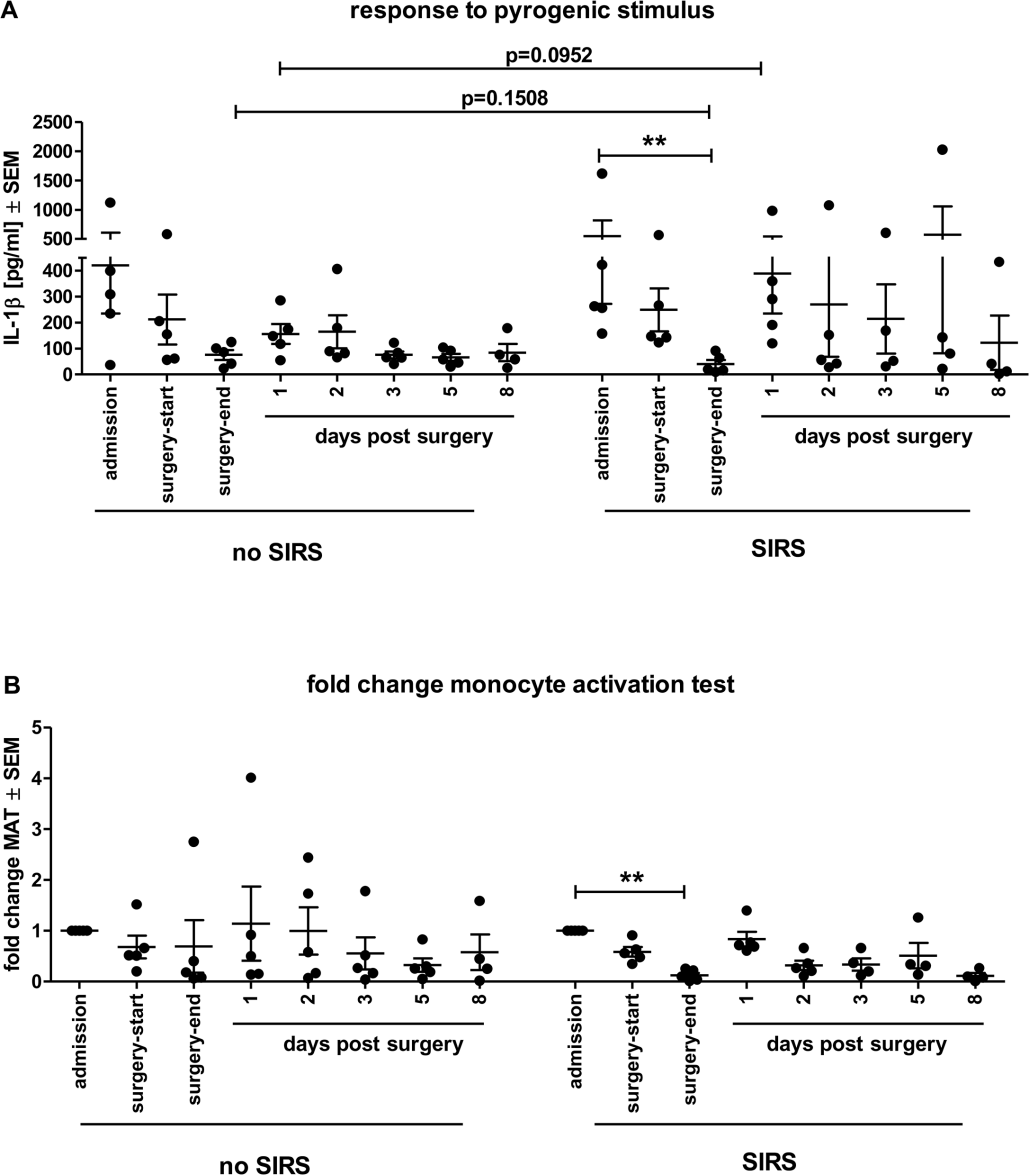
Monocyte activation test (MAT) revealed reduced responsiveness in SIRS patients. Cryopreserved blood of each patient at individual time points was stimulated with reference endotoxin (0.5 EU/ml) corresponding to 0.04 EU/ml in the actual incubation for 8 h at 37°C 5% CO_2_. (A) Individual stimulation responses (shown as IL1β concentrations ± SEM) of diluted cryopreserved patient blood to an LPS stimulus show a tendency towards decreased responses (p = 0.1508) at the end of surgery but increased responses one day after surgery (p = 0.0952) in the SIRS group; Mann Whitney U-test. (B) When the responses to the LPS stimulus are normalized to the responses on the admission day, there is a significant decrease at the end of surgery obvious in the SIRS but not in the control group. ** p < 0.01 Kruskal-Wallis with Dunn’s multiple comparison post hoc test for selected data pairs.

The monocyte activation test (MAT) was developed for human specific pyrogen detection and is based on the reaction of human monocytic cells towards the presence of pathogen-associated molecular pattern (PAMP) [25]. This reaction, measured via the production of inflammatory cytokines, is therefore also an indicator of functional, responsive monocytes. In sepsis conditions, the ability of monocytes to be stimulated has been reported to decrease resulting in dysfunctional states and anergy [26,27]. Here, the response to *ex vivo* endotoxin stimulation was investigated with cryopreserved patient whole blood in order to identify signs of anergic monocytes. All 8 time point samples per patient were investigated in one assay. The stimulation with increasing LPS concentrations resulted in sigmoidal response-curves. All patient samples could be stimulated but the height of responses varied greatly. Since the validated MAT test format specifies an endotoxin contamination limit of 0.5 EU/ml that corresponds to the equivalent rise in rabbits’ body temperature in the rabbit pyrogen test [25] this concentration was chosen to compare secreted IL1β concentrations between time points and patient groups. In both groups, SIRS and control, a decrease in IL1β responses to LPS can be observed at the end of surgery, but only in SIRS patients was this significantly lower than the response at admission (Fig 4A responses and 4B fold changes of the responses). This loss of response at the end of surgery could not be attributed to lower leukocyte counts, since those numbers tended to increase rather than decrease (see Fig 1E). Between SIRS and control group, the responses were not significantly different (p = 0.1508) but this trend towards lower responses could potentially be verified in a larger cohort.

## Discussion

Patients suffering from systemic inflammatory response syndrome (SIRS) due to bacteria (sepsis) or other sterile stressors face a highly increased mortality rate, especially when treatment is not initiated promptly [20]. The symptoms of systemic inflammation as defined by the ACCP/SCCM consensus conference are initially very similar in sepsis and sterile SIRS (see introduction [3]) despite the very heterogeneous nature of causes. This classification has recently been challenged. Some researchers called for a change in the “two-or-more-symptoms” rule, on the one hand because there is no discrete transition point in severity when two symptoms are met and on the other hand because a small subgroup of SIRS-negative septic patients can be missed [28]. A higher predictive value for the outcome of SIRS cases was ascribed when two symptoms persisted longer than six hours or when three or more criteria were met [9]. Originally derived from staging cancer progression, a different model for SIRS classification was proposed [29]; reviewed in [30]. This PIRO (Predisposition, Insult, Response of host, Organ dysfunction) model however, was described as being useful for exclusion/inclusion criteria for clinical studies, but was not judged as robust model, especially lacking criteria for interventions [31]. In this preliminary study, we therefore complied with meeting two or more SIRS criteria as is used in most current studies. The concept of PIRO in terms of looking at predisposition of patients and the insult leading to SIRS was followed in our study by selecting patients scheduled for elective (not emergency) surgery that overall had similar characteristics (such as age, height) and comorbidities very common to patients with heart diseases such as arterial hypertension and adipositas. Moreover, the insult potentially leading to development of SIRS was also similar for the whole patient cohort as patients received the same anesthetics and surgery was performed with the use of CPB. The potential effect of statins (commonly taken by patients with hyperlipidemia) on development of SIRS after CPB is still controversial with reports decreasing the risk [32] and having no effect [33].

By selecting known sensitive inflammatory makers (such as pro- and anti-inflammatory cytokines and CRP, PCT) that were the basis of many investigations and judged useful to predict developing SIRS/sepsis or distinguish between categories, we aimed on establishing the usefulness of those markers in the context of cardiovascular surgery, especially as very early predictive markers before SIRS severity increases. Together with currently discussed cell-activation markers (sTREM-1, ESM-1, cfDNA, monocyte responsiveness) we wanted to establish differences in the immune status of the cardiac surgery patients that have an uneventful recovery and those that develop SIRS/sepsis for timely intervention.

We could show that acute phase proteins CRP and PCT as well as IL6 and IL8 increased after surgery but to similar extends in both SIRS and control groups. TNFα plasma concentrations only showed marginal increasing tendencies at the end of surgery, but also equally in both groups. Those often used sepsis biomarkers were thus not helpful predicting SIRS in our study. Such missing correlation in SIRS patients was not unexpected since the very sensitive marker CRP is upregulated fast (within 2 h) in response to inflammation in general [34], and an association for developing coronary heart disease was also attributed to increased CRP levels [35], hence this likely is an inherent phenomenon in our whole study group. Similarly, PCT the most widely utilized “new generation” biomarker that, especially in combination with additional inflammatory markers such as IL6 and CRP, is reported in some studies as having a good predictive value for sepsis [36–38], was temporarily increased after surgery in both groups. The lack of significant differences between the SIRS and control group likely reflects the common response to stressors of undergoing surgery in the whole study group and such slight elevations were previously reported in SIRS [38]. Similar observations of temporary increases were made with most pro-inflammatory cytokines. While the combination of PCT and IL6 yields a better prognostic value for sepsis, with IL6 serving to monitor effectiveness for antibiotic treatment [39], lower levels are found in SIRS compared to sepsis patients [40]. This was also seen in our study, where IL6 and IL8 were increased but not significantly different between groups, thus not able to distinguish which patient would likely develop SIRS after surgery. An exemption was the pro-inflammatory cytokine IL1β that was markedly increased in SIRS patients directly after surgery compared to their admission values. As individual IL1β responses after stressors vary considerably in humans [22] we specifically looked at individual increases compared to admission times. Here, a significant increase was only seen in SIRS group. To our knowledge, this observation is unique and can together with additional markers well serve early identification of SIRS. In the literature, IL1β is also controversially discussed; on the one hand it was not only increased in septic patients but was also a good predictor for early mortality within 48 h [41], on the other hand a negative evaluation of the relationship between many inflammatory cytokines and sepsis was reported [42]. As IL1β is a cytokine with polyvalent effects, e.g. increasing expression of almost all other cytokines, adhesion molecules, expression of tissue proteases and because of its stimulatory effects myeloid progenitor cells may induce neutrophilia [43], this observation may be a key event in dysregulation of the immune system during SIRS. Together with the different time-frame in counterregulation by the anti-inflammatory cytokine IL10 in our SIRS patients, this result suggests that already at the end of surgery an imbalance of pro- and anti-inflammatory cytokines is initiated eventually contributing to the development of SIRS symptoms. While higher blood IL10 levels paralleled the severity of septic shock [44], no clear differentiation was possible between SIRS and sepsis on the basis of IL10 alone [45]. This appears to be similar in our study, where the tendency of reduced IL10 concentrations can be used augmenting the discrimination on the basis of IL1β increases in SIRS patients. Calculating the ratio of individual IL1β/IL10 levels, those tended to be higher in SIRS patients compared to the control group at the end of surgery and one day post surgery. Furthermore, the decreased responsiveness of SIRS patients’ blood monocytes to an endotoxin challenge at the end of surgery, suggesting anergy of the cells [27], together with the tendency to stronger responses than the control group one day later, underlines the tendency of early dysregulation of the immune system.

The endothelial cell secreted protein endocan (ESM-1) that is upregulated in response to pro-inflammatory cytokines TNFα and IL1β [46,47] was only increased in the control but not in SIRS patient group. This latter observation contrasts with the increased IL1β levels in this study’s SIRS group and reports that ESM-1 increase was a marker of respiratory failure during sepsis [48]. It may well be that the observed increased IL1β levels alone were not sufficient to highly upregulate that particular maker or that other anti-inflammatory mechanisms counteracted the endothelial activation. This is congruent with the observation that ESM-1 levels are strongly upregulated only at later stages of severe sepsis and organ dysfunction [49]. The transient increase of this marker likely reflects the activation of the endothelial cells due to tissue injury during surgery; however, the higher levels in the control group are interesting in itself and could be an additional indication of an imbalanced regulation in beginning SIRS.

We also investigated two receptors expressed on phagocytes that are involved in pattern recognition and inflammatory responses: the triggering receptor expressed on myeloid cells (TREM)-1 and CD163. TREM-1 is described as amplifier of inflammatory responses stimulating the release of pro-inflammatory cytokines and upregulating surface expression of cell activation markers [50]. Activated phagocytes release the receptor that can be found as soluble form in plasma, suggested as a sort of decoy for the natural ligand during infection preventing further activation of the cells [51]. An increase in soluble TREM-1 (sTREM-1) has been described for sepsis patients, where levels could predict survival rates in sepsis better than PCT or CRP [52], were a good assessment for the prognosis of positive blood cultures [53] and noted as reliable biomarker for sepsis [54]. While TREM-1 surface expression on monocytes and neutrophils in other non-infectious inflammatory disorders was described as hardly detectable [50], a more recent study could reveal that surface levels of TREM-1 increase in all inflammatory conditions, also in SIRS [55]. However, soluble TREM-1 was not investigated in those studies. To our knowledge the increased sTREM-1 plasma levels observed in our study in SIRS patients directly at the end of surgery compared to control group is the first description of this phenomenon. This observation is congruent with the described stronger IL1β secretion in SIRS patients that may be due to amplification of the inflammatory response by TREM-1 on the phagocytes. Together, this points to a stronger and less well controlled immune activation in SIRS patients than in the control group after surgery. The consistently, albeit not significantly, higher IL10 concentrations in SIRS patients from day two post surgery also match a derailed immune response common to SIRS. This again is in line with the increasing trend in plasma levels of soluble CD163 (sCD163) in SIRS patients at the end of surgery. This scavenger receptor, expressed on monocytes/macrophages, is involved in clearance of free hemoglobin by binding of the hemoglobin-haptoglobin complexes [56] that induce IL10 secretion [57,58], but expression is in turn also upregulated by IL6 and IL10 [59]. Shedding of the molecule from cell surface is initiated upon activation of extracellular Toll-like receptors (TLR) [60]. Increased plasma sCD163 was linked to a good prognostic assessment of sepsis severity [54] and described significantly upregulated in (severe) sepsis compared to SIRS [61], whereas a study in Denmark reported lacking discrimination between infected and non-infected patients [62]. Studies specifically investigating cardiovascular surgery patients however, determined increased sCD163 levels in patients undergoing surgery with ECC 4 h after the start of surgery [63] or 1 h post de-clamping [24]; a time-frame congruent with the slightly increased levels at the end of surgery observed in our study. The reason for the merely minor elevation in sCD163 could be a) the degree of monocyte activation was not significantly different from the no-SIRS group or b) that differences were not prominent due to patients’ older age [64]. However, this study did not directly measure monocyte CD64 levels, so as to understand cellular and soluble levels, which may be required for a more detailed understanding of the true state of monocyte activation.

Another novel biomarker discussed for inflammatory conditions is the occurrence of cell-free DNA (cfDNA) in plasma. The presence of cfDNA in plasma was already described decades ago [65], 1948 reviewed in [66], but only recently the source and mechanism was attributed to neutrophils that cast out their DNA and histones together with antimicrobial peptides as defense mechanism triggered by bacteria (reviewed in [67,68]) or mitochondrial DNA [69] that itself may evoke SIRS [70]. Here, we observed a significant elevation of cfDNA levels in the non-SIRS control group at the end of surgery compared to the levels at admission. Interestingly, this increase was lower and not significant in SIRS patients. The majority of studies investigating cfDNA as a potential sepsis biomarker have enrolled sepsis, severe sepsis or even patients with MODS. In those studies, the significant elevation of plasma cfDNA or mitochondrial DNA [71,72] were predictive for disease severity and survival [73–76]. These outcomes are in line with an observation made by Margraf and colleagues that DNase levels are reduced in patients developing sepsis and organ dysfunction [77]. On the other hand, a study conducted by Garnacho-Montero and co-workers found no significant difference in cfDNA levels between SIRS and sepsis [78]. Since a rise in cfDNA is also seen in patients with coronary heart disease [79] the increase seen after surgery in non-SIRS control group in our study likely reflects the tissue trauma during surgery, similar to the endocan levels, but does not help to indicate beginning SIRS. A control sample from a SIRS patient non-survivor introduced into the cfDNA assay revealed highly elevated cfDNA levels indicating that in general cfDNA has the potential to serve as a SIRS predictive biomarker but not at very early stages.

We also noted that 2 of 5 SIRS patients did have higher levels of sCD163, cfDNA and sTREM-1, which correlated with the clinical observation of a prolonged increase in heart rate and respiratory rate over the observation period. This indicates that a further study in a larger cohort of patients with differing clinical outcomes might more clearly define the diagnostic utility of such new biomarkers of SIRS or sepsis.

Overall, individualizing the cytokine responses over patients’ admission values revealed a distinctive significant increase of IL1β in SIRS patients already at the end of surgery. Together with increased sTREM-1 concentrations and the reduced responsiveness to an endotoxin stimulus, those markers form a unique combination and may identify patients at risk of developing SIRS after cardiovascular surgery. This however, needs verification in a larger patient cohort that is planned in due course in our hospital.

## Conclusions

In this small scale prospective study in patients undergoing cardiovascular surgery, we could show that the widely used inflammatory markers such as CRP and IL6 were elevated after surgery, but could not predict developing SIRS. However, the combination of those markers with individual patients’ changes in IL1β and IL10 plasma concentrations as well as soluble TREM-1 levels and the responsiveness towards an endotoxin stimulus can provide valuable information for early SIRS prognosis. To our knowledge, this is the first description linking this particular combination of biomarkers to development of SIRS after cardiovascular surgery. Because of the small sample size in this study, the prognostic value of those markers have to be investigated in a larger patient cohort to gain stronger statistical power. Nevertheless, analysis of these predictive biomarker combination may help to faster identify beginning SIRS and enable a timely therapeutic intervention to counteract the escalating immune mechanisms.

## Supporting Information

S1 Fig Plasma levels of C-reactive protein (A) and procalcitonin (B) have low predictive value for early prognosis of SIRS. Rising plasma concentrations of both markers (CRP and PCT) can be measured on days 1 and 2 post surgery with a subsequent slow decrease thereafter in both SIRS and control group. Only the increase in PCT is significant at day 1 post surgery (* p <0.05; Kruskal- Wallis with Dunn’s multiple comparison post-hoc test) but equally within both SIRS and control group. Shown are mean plasma concentrations ± SEM. Dotted lines indicate threshold to pathological ranges.
(TIF)

S2 Fig Patient plasma cytokine levels at the indicated sampling time points. (A) IL1β (B) IL6 (C) IL8 (D) IL10 (E) TNFα. Only IL6 an IL8 show a significant increase in plasma levels at the end of surgery within the SIRS and control group. Shown are mean cytokine concentrations ± SEM. *** p <0.001; Kuskal-Wallis with Dunn’s multiple comparison post-hoc test for selected data pairs. No differences were observed between the two groups.
(TIF)

S3 Fig Plasma levels of endocan (A), soluble CD163 (B) and cfDNA (C) of SIRS and aseptic patients at the indicated sampling time points. No significant difference in plasma levels of endocan, cfDNA and sCD163 markers could be observed between SIRS and control group. Endocan and cfDNA were significantly increased in control patients at the end of surgery, but not in SIRS patients. Shown are mean plasma concentrations ± SEM. * p < 0.05 Kruskal-Wallis with Dunn’s multiple comparison post-hoc test for selected data pairs.
(TIF)
