# Supplementary figures and images for: Identification of Predictive Early Biomarkers for Sterile-SIRS after Cardiovascular Surgery
Source: PLoS One. 2015 Aug 11;10(8):e0135527. doi: 10.1371/journal.pone.0135527 (PMC4532358; doi:10.1371/journal.pone.0135527)

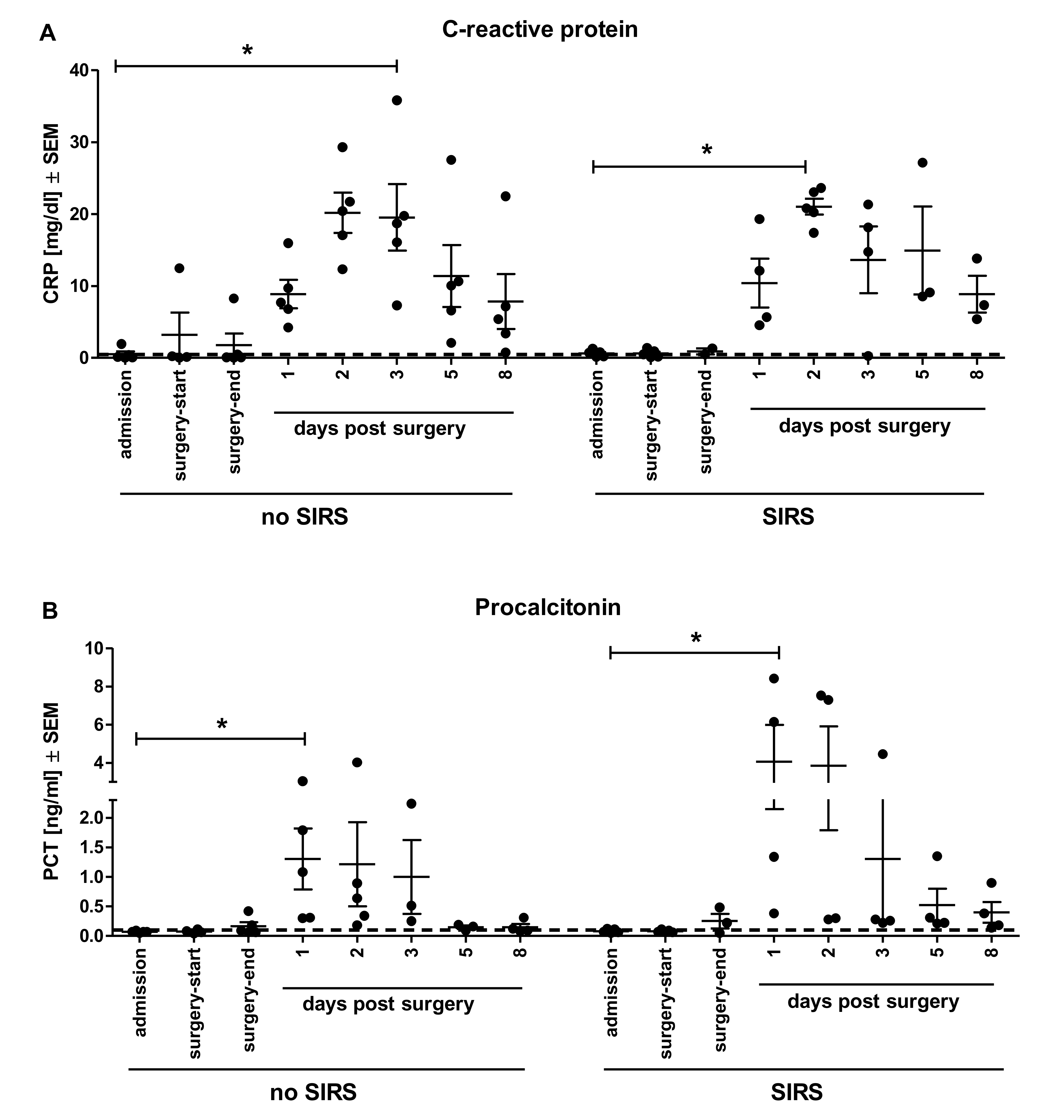

Supplement: S1 Fig — Rising plasma concentrations of both markers (CRP and PCT) can be measured on days 1 and 2 post surgery with a subsequent slow decrease thereafter in both SIRS and control group. Only the increase in PCT is significant at day 1 post surgery (* p <0.05; Kruskal- Wallis with Dunn’s multiple comparison post-hoc test) but equally within both SIRS and control group. Shown are mean plasma concentrations ± SEM. Dotted lines indicate threshold to pathological ranges. (TIF) [file pone.0135527.s001.tif]

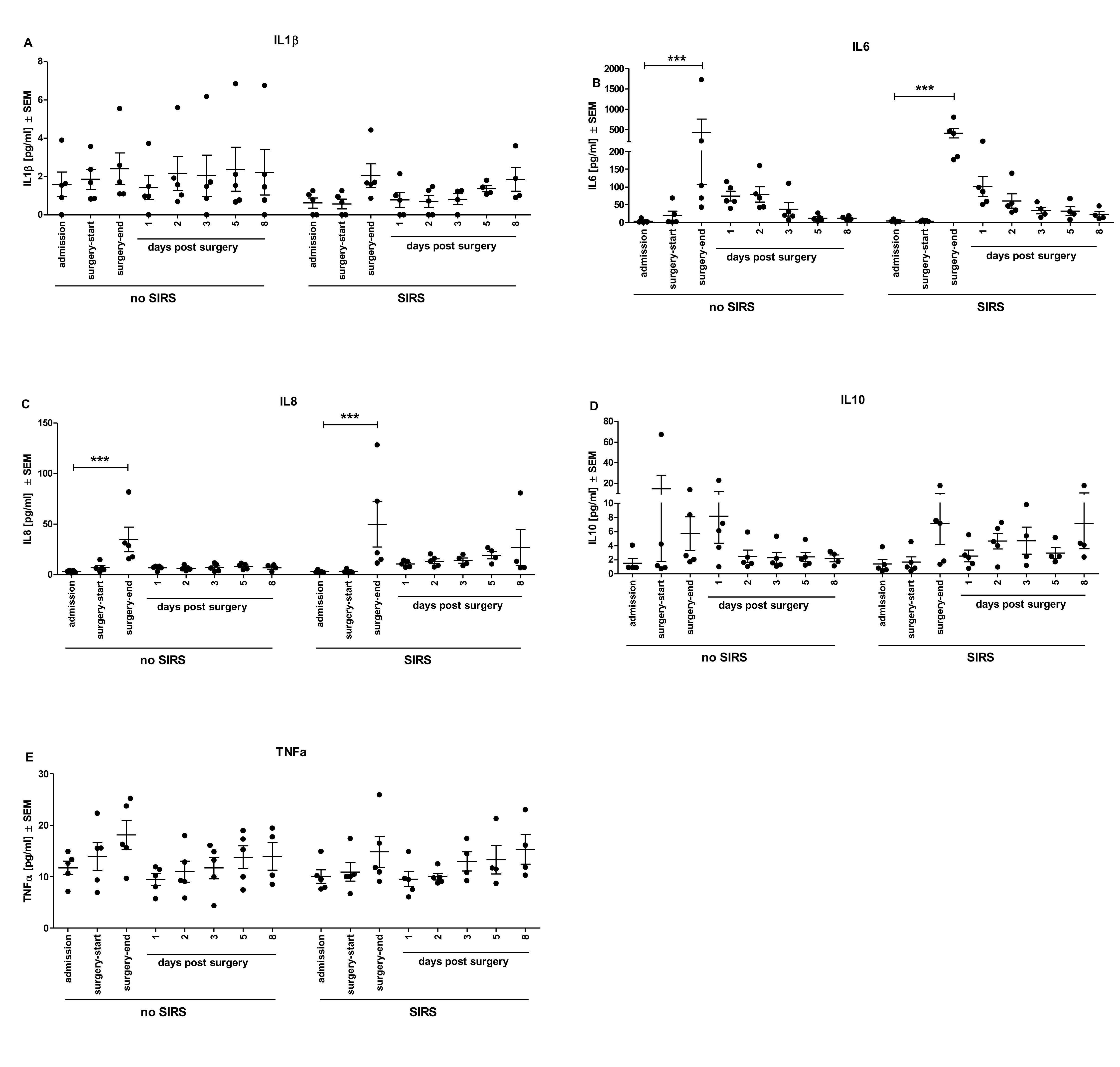

Supplement: S2 Fig — (A) IL1β (B) IL6 (C) IL8 (D) IL10 (E) TNFα. Only IL6 an IL8 show a significant increase in plasma levels at the end of surgery within the SIRS and control group. Shown are mean cytokine concentrations ± SEM. *** p <0.001; Kuskal-Wallis with Dunn’s multiple comparison post-hoc test for selected data pairs. No differences were observed between the two groups. (TIF) [file pone.0135527.s002.tif]

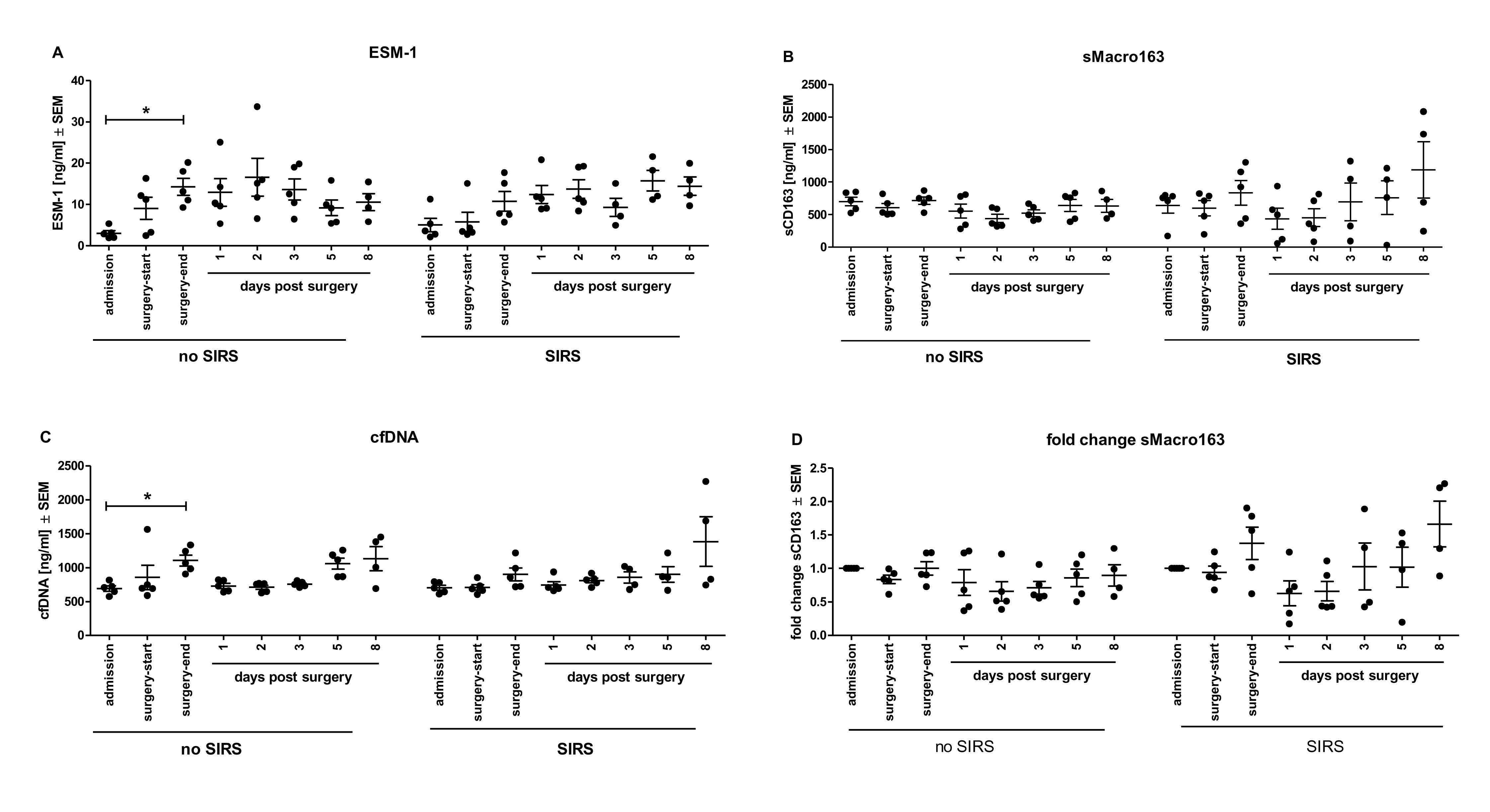

Supplement: S3 Fig — No significant difference in plasma levels of endocan, cfDNA and sCD163 markers could be observed between SIRS and control group. Endocan and cfDNA were significantly increased in control patients at the end of surgery, but not in SIRS patients. Shown are mean plasma concentrations ± SEM. * p < 0.05 Kruskal-Wallis with Dunn’s multiple comparison post-hoc test for selected data pairs. (TIF) [file pone.0135527.s003.tif]
